# The emerging roles of Notch signaling in leukemia and stem cells

**DOI:** 10.1186/2050-7771-1-23

**Published:** 2013-07-18

**Authors:** Na Liu, Jingru Zhang, Chunyan Ji

**Affiliations:** 1 Department of Hematology, Qilu Hospital, Shandong University, 107 West Wenhua Road, Jinan, Shandong 250012, P. R. China

## Abstract

The Notch signaling pathway plays a critical role in maintaining the balance between cell proliferation, differentiation and apoptosis, and is a highly conserved signaling pathway that regulates normal development in a context- and dose-dependent manner. Dysregulation of Notch signaling has been suggested to be key events in a variety of hematological malignancies. Notch1 signaling appears to be the central oncogenic trigger in T cell acute lymphoblastic leukemia (T-ALL), in which the majority of human malignancies have acquired mutations that lead to constitutive activation of Notch1 signaling. However, emerging evidence unexpectedly demonstrates that Notch signaling can function as a potent tumor suppressor in other forms of leukemia. This minireview will summarize recent advances related to the roles of activated Notch signaling in human lymphocytic leukemia, myeloid leukemia, stem cells and stromal microenvironment, and we will discuss the perspectives of Notch signaling as a potential therapeutic target as well.

## Introduction

The Notch signaling pathway is highly conserved from Drosophila to human and plays an important role in the regulation of cell proliferation, differentiation and apoptosis [1]. Moreover, it has been suggested that Notch signaling may be responsible for the development and progression of human malignancies, including leukemia.

### Notch signaling pathway

#### Notch and the ligands

Four members of Notch proteins have been identified to date in mammals, including Notch1-4 [2-5]. The Notch proteins are single-pass transmembrane receptors, which are composed of extracellular, transmembrane and intracellular domains. The extracellular domain of all Notch proteins contain epidermal growth-factor-like repeats (EGFLR) and three LIN Notch (LNR) repeats, whereas the intracellular domain consists of the RAM23 domain (RAM) and seven Ankyrin/CDC10 repeats (ANK), necessary for protein-protein interactions. Moreover, five canonical Notch ligands have been found in mammals: Dll1 (Delta-like 1), Dll3 (Delta-like 3), Dll4 (Delta-like 4), Jagged1 and Jagged2 [2-5]. Notch ligands are transmembrane proteins of which the extracellular domain contains a characteristic number of EGF-like repeats and a cysteine rich N-terminal DSL domain, responsible for the interaction with Notch receptors.

#### Notch signaling activation

Notch signaling has been shown to be initiated by binding of the Notch transmembrane receptors with their specific ligands between two neighboring cells [6]. Upon activation, Notch receptors undergo a cascade of metalloprotease tumor necrosis factor-α-converting enzyme (TACE) and γ-secretase complex proteolytic cleavages, releasing the Notch intracellular domain (NICD). Subsequently, the NICD translocates into the nucleus and interacts with the DNA binding protein CSL to regulate gene expression. To date, only a few target genes have been identified. The best-known Notch target genes are two families of basic helixloop helix transcription factors: Hes (Hairy enhance of split) and Hey (Hairy/enhancer of spit related with YRPW motif) family [7]. Hes and Hey proteins are helix-loop-helix transcription factors that function as transcriptional repressors. Additionally, target genes of the Notch signaling pathways also include cyclin D1, c-myc, p21, p27, Akt, mTOR, VEGF, etc., some of which are dependent on Notch signaling in multiple tissues, while others are tissue specific [8-21] (Table 1). Nevertheless, many target genes of Notch signaling remain to be determined [8].

**Table 1:** Target genes of the Notch signaling pathways

**Gene**	**Role**	**Tissues**	**Comments**
cyclin D1, cyclin A , p21, p27,	Cell cycle regulators	Hepatocellular cancer, renal cancer	[9,10]
c-myc, NF-κB2 , Akt, mTOR,	Cell proliferation and survival	Keratinocytes, liver, T-ALL,	[11-16]
Hes1, Hes6	Embryonic development	Embryonic neural progenitor cell, human pluripotent stem cells	[17,18]
VEGF, VEGFR-2	Angiopoiesis	Osteosarcoma, endothelial and neural cells.	[19,20]
MMP-9, MMP-2	Invasion and metastasis	Osteosarcoma, pancreatic cancer	[19,21]

### Notch signaling in lymphocytic leukemia

#### T cell lymphocytic leukemia

It has been shown that Notch signaling is abnormally regulated in many human malignancies [22,23]. Notch1 mutations causing Notch signaling continuously activated have been found in nearly 60% of T cell acute lymphoblastic leukemia (T-ALL) patients, making Notch1 the most prominent oncogene specifically involved in the pathogenesis of T-ALL [24,25]. The characterize mutations occur mostly in the heterodimerization (HD) domain and proline, glutamic acid, serine, threonine-rich (PEST) domain of the Notch1 receptor. HD domain mutation leads to a COOH-terminally truncated NICD, whereas PEST domain mutation results in loss of the negative regulatory domain, escaping from FBXW7-mediated degradation and prolongation of the half-life of NICD [26]. Notch1 mutations have been shown to be an early, prenatal genetic event in T-ALL patients [27]. In murine models of T-ALL, Notch1 activation is responsible for directly inducing leukemia and collaborating with other initiating genetic events to perpetuate leukemic growth [28,29]. Moreover, our previous study has shown that Notch1 signaling is also required for hypoxia-induced proliferation, invasion and chemoresistance in T-ALL, suggesting that pharmacological inhibitors of Notch1 signaling may be attractive interventions for T-ALL treatment [30].

Additionally, other Notch signaling and target genes are also involved in the initiation and progression of T-ALL. It has been reported that Notch3 and Hes1 are highly expressed by T-ALL cells, as well as dramatically reduced or absent in remission [31]. Downregulation of Notch3 by small hair RNA (shRNA) has been found to suppress the activity of Notch signaling, leading to growth inhibition and apoptosis induction of T-ALL cells [32].

#### B cell lymphocytic leukemia

Interestingly, the function of Notch signaling in leukemogenesis has been shown to be either oncogenic or tumor suppressive, and it could be context dependent [33,34]. Notch signaling and target genes have been demonstrated to be tumor suppressive rather than oncogenic in a limited number of leukemia types, including B-ALL (Table 2). It has been reported that in contrast to T-ALL, Notch3, Jagged1, Hes2, Hes4 and Hes5 were frequently hypermethylated in B-ALL, associated with gene silencing [33]. Furthermore, restoration of Hes5 expression by lentiviral transduction could give rise to growth arrest and apoptosis in Hes5 negative B-ALL cells but not in Hes5 expressing T-ALL cells [33]. Other investigators confirmed the fact and showed that activated forms of the 4 mammalian Notch receptors (NICD1-4) or hes1 was responsible for growth inhibition and apoptosis enhancement in both murine and human B-ALL [35-37].

**Table 2:** Notch in B cell Lymphocytic leukemia

**Gene**	**Role**	**Leukemia types**	**Comments**
Notch1	Tumor suppressor	B-ALL	[35]
Notch2	Oncogene	B-CLL	[37-39]
Notch3, Notch4	Tumor suppressor	B-ALL	[33,40]
Hes1	Tumor suppressor	B-ALL	[41]
Hes5	Tumor suppressor	B-ALL	[33]

In contrast with B-ALL, Notch signaling could maintain B cell chronic lymphoblastic leukemia (B-CLL) cell survival and apoptosis resistance, undoubtedly indicating an oncogenic role in B-CLL. Emerging evidence suggests that the Notch signaling network is frequently deregulated in human B-CLL with up-regulated expression of Notch1 and Notch2 as well as their ligands Jagged1 and Jagged2 [42]. Moreover, Notch signaling inhibition by the gamma-secretase inhibitors (GSIs) and the specific Notch2 down-regulation using small interfering RNA (siRNA) could promote B-CLL cell apoptosis [38,42]. It has been also reported that Notch2 is not only overexpressed in B-CLL cells but also might be related to the failure of apoptosis-oriented treatment for this disease and deregulation of Notch2 signaling is involved in the aberrant expression of CD23 in B-CLL [39-41]. Taken together, these results suggest that Notch signaling is constitutively activated in B-CLL cells, and can sustain the survival of these cells.

#### Notch signaling in myeloid leukemia

Knowledge about the role of Notch signaling in acute myeloid leukemia (AML) is equally poorly understood. Very recently, Jagged1 and Dll1 were shown to be expressed at significantly higher levels in acute promyelocytic leukemia (APL) samples compared with all other subtypes, as well as normal myeloid populations [43]. Inhibition of Notch signaling by GSIs could reduce self-renewal and colony formation of Kit^+^Lin^-^Sca1^+^ cells from pre-leukemic Ctsg^-^PML-RARA mice [43]. Our previous study has also demonstrated that Dll4 and Notch1 expression were significantly higher in untreated AML patients than in the normal controls, and provides evidence that the activation of Notch signaling may indicate an unfavorable prognosis in AML [44]. These data suggest that Notch signaling can promot AML development [45]; however, other studies have shown opposite function of Notch signaling in AML (Table 3). A significant decrease in the levels of the Notch ligand and activated receptors as well as target genes was reported to be lower in AML samples than in normal hematopoietic stem cells (HSCs), suggesting that Notch signaling is not activated in AML [46-48]. Kannan *et al.* have found that all four Notch homologues and Hes1 were sufficient to inhibit the growth and induced caspase-dependent apoptosis of AML, which were associated with B cell lymphoma 2 (BCL2) loss and enhanced p53/p21 expression [45]. Additionally, the dnMAML (a pan-Notch inhibitor) could not affect AML proliferation *in vitro* but lead to dramatic increases in leukemia burden in two xenograft mouse models, which was associated with p53 dysregulation [45]. The 17-aa peptide with Notch agonist activity was able to activate Notch signaling to induce apoptosis of AML cells [45,49,50]. Besides inducing apoptosis, the recombinant Notch ligand proteins, Dll1 and Dll4 could alter AML blast cells into macrophage-like cells morphologically and increase the expression of differentiation markers such as CD13 or CD14 [51]. Tohda *et al.* also found that the Notch ligands tended to induce differentiation under the specific conditions rather than promoted the self-renewal capacity of AML cells [52]. Overall, different researchers and experiment methods come to different conclusions, illustrating the highly context-dependent nature of the pathway. Due to the complexity of the Notch pathway and limited tools to specifically modulate the this pathway, the function of this signaling is still unclear, and additional studies are needed to clarify the role of various Notch receptors in AML.

**Table 3:** Notch in myeloid leukemia

**Leukemia types**	**Role**	**Mechanism**	**Comments**
Non-APL AML	Tumor suppressor	B cell lymphoma 2 (BCL2) loss and enhanced p53/p21 expression	[45]
APL	Oncogene	Jagged1 and Dll1 were overexpressed. GSIs could reduce self-renewal and colony formation of Kit + Lin-Sca1+ cell	[43]
CMML	Tumor suppressor	Notch1–3−/− or Ncstn−/− mice developed CMML-like disease	[53]
CML	Tumor suppressor	Inhibition of proliferation	[54,55]

Notch signaling appears to play a tumor suppressive role in chronic myeloid leukemia (CML). It is reported that overexpression of the active form of Notch1 or Notch2 in K562 cells resulted in the inhibition of proliferation, accompanied by increased Hes1 mRNA level [54,55]. On the other hand, attenuation of Notch signaling by overexpression of a dominant-negative RBP-J calledRBP-JR218H led to the increased proliferation of K562 cells. Moreover, activation of Notch signaling was found to inhibit the colony-forming activity of K562 cells while repression of Notch signaling played the opposite role [55]. These results provide evidence that Notch signaling might play a role as a tumor suppressor in CML.

#### Notch signaling in leukemia stem cells

Leukemia stem cells (LSCs) arise either from corrupted HSCs or from more differentiated and committed progenitors that acquire self-renewal potential [56-58]. Therefore, targeting this unique property of LSCs—self-renewal capacity—is thought to be a promising way to eradicate disease if one can determine which pathways are critical for LSC, but not HSC. Notch signaling is active in HSCs *in vivo* and downregulated as HSCs differentiated. Inhibition of Notch signaling could lead to accelerated differentiation of HSCs *in vitro* and depletion of HSCs *in vivo*[59,60]. Furthermore, Notch1 drives cell fate decision (the choice between TCRγ/δ orα/β and between CD4^+^ or CD8^+^) by inductive interactions from thymic stromal cells [61,62], suggesting that Notch1 expression is finely regulated during T-cell lineage development [63]. Notch1 is also reported to plays a role in rescuing T cells from apoptosis [64].

**Figure 1:**
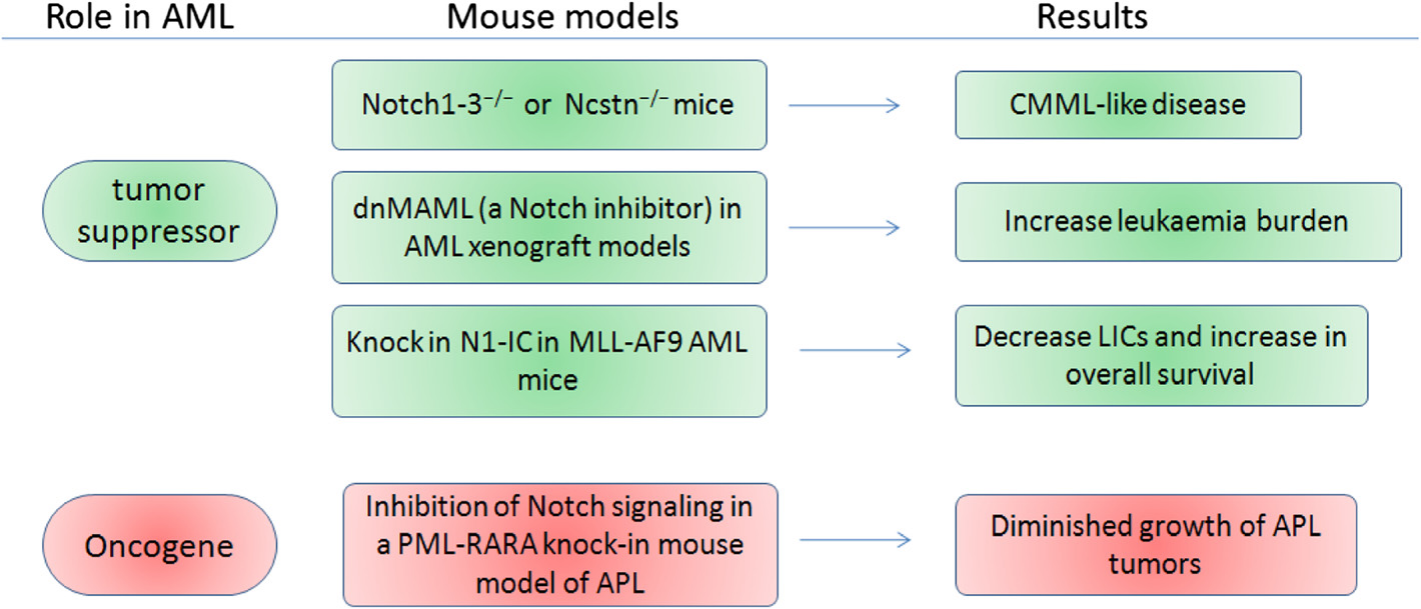
**Mouse models reveal dual roles for Notch receptors.** Mouse models reveal that Notch receptors either promote or inhibit AML development depending on the context.

To date, the role of Notch signaling in LSCs has not yet been examined adequately and seems to be context dependent. Notch signaling was shown to be silenced in CD34^+^/CD38^-^ stem/multipotential progenitor populations from AML patients compared to normal CD34^+^ stem cells. Recently, inactivating mutations of Notch signaling have been described in patients with chronic myelomonocytic leukemia (CMML) [53]. *In vivo* studies have also revealed both oncogenic and tumor suppressive functions for Notch signaling (Figure 1). In an MLL-AF9–induced mouse AML model, Notch signaling was inactive in CD34^+^/CD38^-^ stem/progenitor cells and upregulation of Notch signaling using genetic Notch gain of function models could result in the proliferation inhibition of this populations. Moreover, *in vitro* activation of Notch signaling using synthetic Notch ligand led to rapid cell cycle arrest, differentiation, and apoptosis of AML-initiating cells [65]. Notch1–3^−/−^ or Ncstn^−/−^ mice was also found to develop an aberrant accumulation of granulocyte/monocyte progenitors (GMP), extramedullary hematopoieisis and the induction of CMML-like disease. Furthermore, ectopic expression of Notch1-IC or Hes1 could suppress the expression of key GM commitment genes such as Cebpα and Pu.1, and the CMML-like disease developing in the Ncstn^−/−^ animals [53]. However, an oncogenic role for Notch signaling has been identified by other groups. Grieselhuber *et al.* reported that Jagged1 was higher in Kit^+^Lin^-^Sca1^+^ cells from pre-leukemic Ctsg-PML-RARA mice, and both genetic and pharmacologic inhibition of Notch signaling abrogated the enhanced self-renewal seen in hematopoietic stem/progenitor cells [43]. Taken together, increasing evidence suggests that Notch signaling is involved in the regulation of self-renewal capacity of LSCs in murine models and human disease, and point out where Notch signaling be uniquely required in leukemia.

#### Activation of Notch signaling by stromal microenvironment

Leukemia cell survival relies on leukemic microenvironment, which is composed of bone marrow stromal cells (BMSCs), endothelial cells and other factors. Accumulating evidence emphasized the importance of Notch signaling in the cross-talk between leukemia cells and their stromal microenvironment. BMSCs were shown to induce upregulation of Notch signaling molecules, such as Notch1, Notch3 and4 or Jagged1/2 and Dll1 [40,66]. Moreover, activation of Notch signaling by stromal microenvironment were necessary for leukemia cell survival by preventing blast cell apoptosis and favoring their reciprocal interactions and cross-talk with bone marrow microenvironment [66-68]. Our previous study reported that Notch-1 activation was induced by coculture with BMSCs and down-regulation of Notch-1 increased cocultured Jurkat cell sensitivity to chemotherapy [40,66]. Florence *et al.* also found that coculture of primary human T-ALL with a mouse stromal cell line expressing the Dll1 reproducibly allowed maintenance of T-LiC and long-term growth of blast cells through rescuing from apoptosis [69]. The molecular mechanisms of apoptosis resistance may be associated with a variety of cytokines, such as IL-7 [70,71], lymphocyte function-associated antigen-1 (LFA-1) and intercellular adhesion molecule-1 (ICAM-1) [71]. Inactivation of Notch signaling resulted in the decrease of leukemia cell survival, either cultured alone or cocultured in presence of stromal cells from normal donors and leukemia patients [40]. In addition, previous *in vitro* studies have demonstrated that endothelial cells enhance proliferation and survival of AML cells [72]. Our study showed a bidirectional cross-talk between endothelial and AML cells that had a promoting effect on endothelial cell function, and elucidated a novel mechanism by which the interplay between AML and endothelial cells promotes angiogenesis through VEGF activation of the Notch/Dll4 pathway [67].

#### Inhibitors of Notch signaling and the potential clinical application

The specific and profound involvement of Notch signaling in various leukemic types makes it an ideal target for pharmacological intervention. Several strategies have been proposed to inhibit or modulate this signaling [73,74]. The most widely used drug to globally inhibit Notch signaling is GSIs, which block the cleavage of Notch at the cell membrane, inhibiting release of the transcriptionally active Notch intracellular domain (NICD) subunit. A lot of clinical research or preclinical testing have focused on testing GSIs in the treatment of leukemia, but the results were initially disappointing (Table 4). It has been reported that RO4929097, one of GSIs, could induce insignificant differences in event free survival distribution compared to control in 0 of 8 (0%) of the evaluable ALL xenografts mice [75]. A phase I clinical trial also showed that MK-0752, another GSIs, had limited antitumor activity in relapsed T-ALL patients [76]. What is more, GSIs are nonspecific and can inhibit Notch signaling in the gut, leading to gastrointestinal toxicity, which also limit its application. However, in an attempt to the clinical application of GSIs, dexamethasone was found to abrogate GSI-induced toxicity in the gut and as well GSIs treatment could reverse glucocorticoid resistance in T-ALL patients [77]. Therefore, these results supported a role for combination therapy with GSIs plus glucocorticoids in the treatment T-ALL. In another attempt to remedy this issue, inhibitory antibodies have recently been synthesized for all Notch receptors. A Notch1-specific antibody significantly induced cell cycle arrest and reduced cell proliferation in T-ALL cells. Moreover, in mouse xenograft T-ALL and colon cancer models, the Notch1-specific antibody could induce significant tumor regression and slowing of growth [74], which would pave the way for new clinical trials to evaluate the efficacy of more selective and less toxic antibody-based therapies. The overwhelming potential of Notch-based cancer treatments cannot be ignored.

**Table 4:** Clinical research of GSIs in the treatment of leukemia

**Test**	**Preclinical study**	**Phase I**
Drug	RO4929097	MK-0752
Methods	8 mice (leukemia models) were used in each control or treatment group.	Six adult and two pediatric patients with leukemia (seven with T-ALL and one with AML) received MK-0752
	Percentages of human CD45+ cells were determined	
Results	No significance in event-free survival [53]	Limited antitumor activity and major gastrointestinal toxicity
Comments	[75]	[76]

## Conclusions

Controversy will remain, as we do not understand the complexity of the Notch pathway and tools to specifically modulate the Notch pathway are still limited. Further studies assessing the levels of Notch activation and inhibition in leukemia still need to be carried out. Further advancement in understanding the molecular events of Notch signaling can potentially lead to further clinical benefit.

## Competing interests

The authors declare no competing financial interests.

## Authors’ contributions

All authors have contributed to data preparation, drafting and revising the manuscripts. All authors have read and approved the final manuscript.
